# Fast adaptation of cooperative channels engenders Hopf bifurcations in auditory hair cells

**DOI:** 10.1016/j.bpj.2022.02.016

**Published:** 2022-02-15

**Authors:** Francesco Gianoli, Brenna Hogan, Émilien Dilly, Thomas Risler, Andrei S. Kozlov

**Affiliations:** 1 Department of Bioengineering, Imperial College London, London, UK; 2 Institut Curie, Université PSL, Sorbonne Université, CNRS UMR168, Laboratoire Physico-Chimie Curie, Paris, France

## Abstract

Since the pioneering work of Thomas Gold, published in 1948, it has been known that we owe our sensitive sense of hearing to a process in the inner ear that can amplify incident sounds on a cycle-by-cycle basis. Called the active process, it uses energy to counteract the viscous dissipation associated with sound-evoked vibrations of the ear’s mechanotransduction apparatus. Despite its importance, the mechanism of the active process and the proximate source of energy that powers it have remained elusive, especially at the high frequencies characteristic of amniote hearing. This is partly due to our insufficient understanding of the mechanotransduction process in hair cells, the sensory receptors and amplifiers of the inner ear. It has been proposed previously that cyclical binding of Ca^2+^ ions to individual mechanotransduction channels could power the active process. That model, however, relied on tailored reaction rates that structurally forced the direction of the cycle. Here we ground our study on our previous model of hair-cell mechanotransduction, which relied on cooperative gating of pairs of channels, and incorporate into it the cyclical binding of Ca^2+^ ions. With a single binding site per channel and reaction rates drawn from thermodynamic principles, the current model shows that hair cells behave as nonlinear oscillators that exhibit Hopf bifurcations, dynamical instabilities long understood to be signatures of the active process. Using realistic parameter values, we find bifurcations at frequencies in the kilohertz range with physiological Ca^2+^ concentrations. The current model relies on the electrochemical gradient of Ca^2+^ as the only energy source for the active process and on the relative motion of cooperative channels within the stereociliary membrane as the sole mechanical driver. Equipped with these two mechanisms, a hair bundle proves capable of operating at frequencies in the kilohertz range, characteristic of amniote hearing.

## Significance

How the inner ear amplifies incident sounds at frequencies of several kilohertz is a key question that has remained unanswered despite decades of research into several candidate mechanisms. Here we model the behavior of hair cells, the sensory receptors of the inner ear, and show that they can undergo oscillatory instabilities called Hopf bifurcations because of the effect of Ca^2+^ on the cooperative opening and closing of mechanotransduction ion channels. As any dynamical system close to the bifurcation point, a hair cell behaves as a nonlinear oscillator that can amplify its input on a cycle-by-cycle basis. We find that our proposed mechanism can operate in the kilohertz range.

## Introduction

The active process of the inner ear is the vibrating soul of the auditory system (1). The ear expends energy to amplify sounds, sharpen its frequency selectivity, and help compress six orders of magnitude in sound amplitude into a hundredfold range in the firing rate of the auditory fibers (2). This process is rooted in the biophysical properties of individual hair cells, the sensory receptors of the inner ear. These cells act as microphones that pick up sound vibrations with their bundles of enlarged microvilli, called stereocilia, and as stimulus amplifiers (3). Signal transduction and amplification rely on elastic molecular filaments, the tip links, which are connected to mechanically sensitive ion channels that open and close in unison with hair-bundle vibrations (4). Signal amplification, powered by the active process, requires a source of energy and has to be fast to operate at physiological frequencies of up to 20 kHz in humans and even higher frequencies in some other mammals.

Although the precise biophysical mechanism at the origin of the active process is still a matter of debate, the ear as a whole, as well as individual hair cells in amphibians, shows signatures of dynamical systems operating close to Hopf bifurcation points, where the system transitions from a quiescent regimen to spontaneous oscillations (5,6). Such dynamic systems display the most salient features of the active ear, including active amplification of low-intensity stimuli, sharp frequency selectivity, compressive nonlinearity in response amplitude, and spontaneous oscillations (2). Spontaneous oscillations have been proposed to underlie spontaneous otoacoustic emissions, the tones that healthy ears emit when in a quiet environment (7), and have been measured in hair bundles of the bullfrog sacculus (8). Despite the unifying power of modeling the ear as a collection of nonlinear oscillators close to Hopf bifurcation points, the connection between the cellular and molecular biology of the inner ear and this overarching mathematical description has not yet been fully understood.

Current models of mechanotransduction reproduce well the behavior of the hair bundles of the bullfrog sacculus, which exhibit all features of the active process as they detect and amplify stimuli at frequencies on the order of tens to a hundred hertz (5,9). These models, however, struggle to explain how auditory hair cells can achieve active amplification at frequencies in the kilohertz range, typical of mammalian hearing. This stems from the fact that such models are limited by the pace of myosin motors on which they rely to reset the working range of the hair cell on a cycle-by-cycle basis (5). Despite several pioneering studies on this subject (9, 10, 11, 12), no microscopic model of hair-bundle motility has so far been able to reproduce the properties of active amplification at frequencies in the kilohertz range with only a minimal number of known, possible states of the channels as well as realistic values of the gating swing—the amplitude of the conformational change of an individual channel upon opening or closing. In mammals, sparse evidence suggests that hair bundles are capable of generating forces that could drive the active process (13,14), but no mammalian hair cell has ever been observed to oscillate spontaneously. The source of amplification in the cochlea has rather been attributed to electromotility, the ability of mammalian outer hair cells to change their length in response to changes in their transmembrane electrical potential (15). Electromotility, however, is constrained by the frequency response of prestin, the molecular driver of electromotility in outer hair cells, which is low pass filtered with a corner frequency of a few kilohertz (16). Measurements *in vivo* indicate that this cutoff frequency lies about 2.8 octaves below the characteristic frequency of the basilar membrane at the same location, suggesting that electromotility is unlikely to be the main mechanism responsible for sound amplification on a cycle-by-cycle basis (17). Although essential for mammalian hearing, electromotility alone cannot account for all hallmarks of the active process because it is mostly linear at small displacements (18) and because the hallmarks of the active process persist under conditions where electromotility vanishes (19). For instance, stimulus amplification in a cochlear segment could be achieved independently from the hair-cell transmembrane electrical potential, the driver of electromotility (19). Finally, birds and some species of lizards can hear sounds in the kilohertz range, but their hair cells are devoid of prestin and do not display electromotility. If there is a parsimonious explanation for amplification, a single mechanism underlying the active process at these frequencies in all amniotes, one must look for it elsewhere.

Here we present a new model of hair-bundle motility that relies on the electrochemical gradient of Ca^2+^ as an energy source to power active oscillations, similarly to what has been proposed by Choe et al. (10). We base our model on the earlier proposal that each tip link is connected to more than one mechanotransduction channels whose states are reciprocally coupled to hair-bundle motion by elastic forces mediated by protein linkages as well as by the membrane (20). This model is not limited by the action of myosin motors, and we show here that it can produce spontaneous oscillations at frequencies of a few kilohertz, characteristic of the hearing organs of birds, some lizards, and large mammals. With two channels per tip link, the model relies on one Ca^2+^ binding site per channel with transition rates derived from thermodynamic principles. Associated with the two different states of the channel pair—open-open or closed-closed—is a difference in the membrane elastic energy because of different membrane deformations to match the different thicknesses of the hydrophobic region of each channel in its open and closed states (20,21). As a result, the model produces spontaneous oscillations with no requirement for a large individual-channel gating swing as long as the hydrophobic region of the channel has different thicknesses in the open and closed states. We describe the details of this model in the next section.

## Model

**Figure 1:**
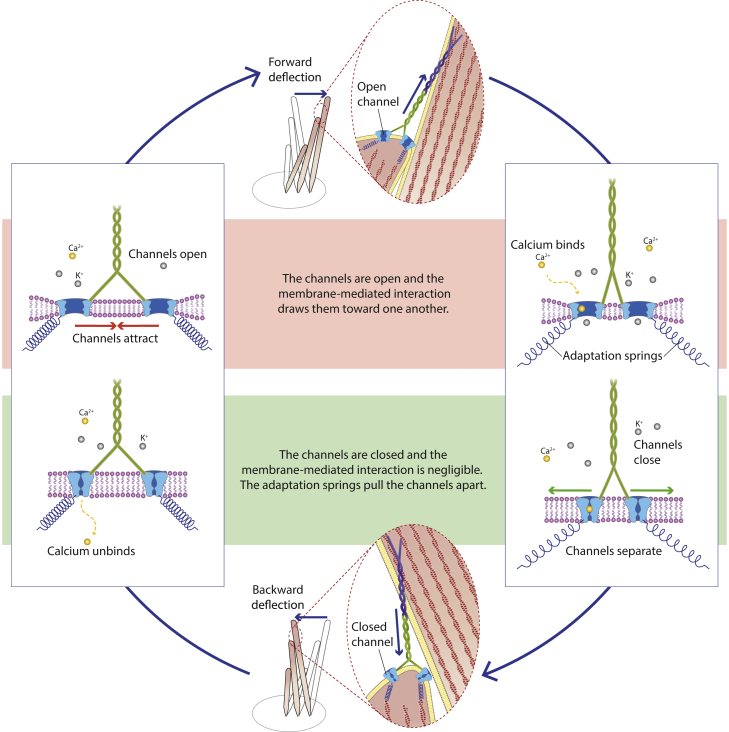
Schematics of the hair-bundle oscillations driven by Ca^2+^ binding and unbinding (bundle deflections are exaggerated for the purpose of the illustration). We start the description of the cycle from the left box, top drawing, with both channels open and no Ca^2+^ bound. In that configuration, the membrane-mediated interaction between the two channels drives them toward one another (left box, top panel, red arrows). This movement relaxes tension in the tip link, allowing the stereociliary pivots to drive the hair bundle to the right (top illustration, blue arrows). The channels being open, Ca^2+^ enters and subsequently binds to one of the two channels, facilitating its closure. Because the OC state is strongly disfavored energetically as a result of the membrane-mediated interaction between the channels, this triggers closure of the second channel almost simultaneously (right box, bottom panel). In this CC configuration, the membrane’s elastic potential is almost flat, and tension in the adaptation springs pulls the channels apart (green arrows). The resulting increase of tension in the tip link causes the bundle to twitch backward (bottom illustration, blue arrows). Now that the channels are closed, the intracellular Ca^2+^ concentration at the binding sites decreases, which lowers their occupancy (left box, bottom panel). Tension in the springs then tends to reopen the channels, bringing us back to where we started and completing the cycle of oscillations. At steady state, a thermodynamic equilibrium exists between the OO state with the channels closer to one another and the CC state with the channels farther apart. To see this figure in color, go online.

**Figure 2:**
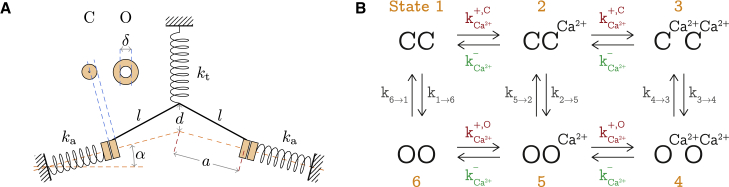
(*A*) Geometry of the two-channel model and associated parameters. The orange annuli represent the mechanosensitive channel in the closed (C) and open (O) configurations, seen orthogonally to the membrane plane. The system of springs is shown in the CC configuration. (*B*) Schematic representation of the six different states considered for a channel pair. States 1, 2, and 3 correspond to both channels closed, with, respectively, zero, one, or two calcium ions bound. States 4, 5, and 6 correspond to both channels open similarly, with different numbers of calcium ions. Each allowed direct transition is characterized by a rate constant, where the transitions 1⇌6, 2⇌5, and 3⇌4 correspond to opening or closing of the channels, and 1⇌2, 2⇌3, 4⇌5, and 5⇌6 correspond to binding or unbinding of one Ca^2+^. To see this figure in color, go online.

Based on previous work (20), we hypothesize that, in a mature hair bundle, every tip link is connected to two functional mechanosensitive ion channels (22,23). These channels are mobile in the membrane, and they each connect to one of the two strands of the tip link at its lower end (24). Two adaptation springs anchor the channels to the cytoskeleton (Figs. 1 and 2 *A*). Although initially a conjecture (20), this connection was later confirmed experimentally in *Caenorhabditis elegans* for transmembrane channel-like proteins (25), which are molecular components of hair-cell mechanotransduction channels (26, 27, 28). The two channels function as a unit because of reciprocal coupling between their conformational states (open or closed) and the local thickness of the membrane (21,29,30). This reciprocal coupling stems from the different hydrophobic regions of the channel in the open and closed states, causing a state-dependent hydrophobic mismatch with the lipid bilayer around the channel and, therefore, deforming it differently in these two states (21,30). Minimizing the energy, which includes the elastic energy of the membrane surrounding the channels, results in cooperative gating so that only two gating states (open-open (OO) and closed-closed (CC)) are effectively at play for the pair. In contrast to the classical gating spring model (4,31), the observed relaxation of the hair bundle upon channel opening is provided by the motion of the channels in the membrane plane rather than by the individual-channel gating swing.

Here we add to that description an active process based on a cycle of Ca^2+^ binding and unbinding to the channels, similar to what was proposed by Choe et al. (10) in 1998. We hypothesize that the binding of one Ca^2+^ ion to a channel lowers the energy of the closed state while leaving the open state unaffected. Given that Ca^2+^ binding is a diffusion-limited process, the rate of Ca^2+^ binding is proportional to its local concentration, whereas that of Ca^2+^ unbinding does not depend on it. Because Ca^2+^ diffusion over several nanometers occurs on much shorter timescales than those of interest here, we estimate that the Ca^2+^ concentration at the channel’s binding site equilibrates instantaneously to two values, one for each channel state (open or closed). We therefore introduce two rates of Ca^2+^ binding, breaking thermodynamic equilibrium. We show that such an active process is able to drive spontaneous oscillations of the hair bundle (Fig. 1). In response to a change of parameter values, and notably of Ca^2+^ concentration in the vicinity of the open channel, the hair bundle transitions between a quiescent and an oscillatory regimen. This transition corresponds to a Hopf bifurcation, where the system’s sensitivity is maximal.

### Force balance

This part of the model is identical to the model published in (20). Force balance on the hair bundle reads as follows:

(1) $$M_{\text{HB}}\ddot{X}=-\xi_{\text{HB}}\dot{X}-K_{\text{sp}}\left(X-X_{\text{sp}}\right)-\gamma \sum_{j=1}^{N_{\text{t}}}f_{\text{t}}^{j}\;.$$

Here, *X* denotes the position of the tip of the hair bundle projected onto the direction of mechanosensitivity, $\dot{X}$ and $\ddot{X}$ are its first and second time derivatives, and $X_{\text{sp}}$ is the resting position of the hair bundle in the absence of tip links because of the action of the stereociliary pivots. $M_{\text{HB}}$ is the hair bundle’s apparent wet mass (32) (see Parameter values), $\xi_{\text{HB}}$ the bundle’s viscous drag coefficient, $K_{\text{sp}}$ the combined stiffness of the stereociliary pivots, and $N_{\text{t}}$ the number of tip links. The scaling factor γ is the approximately constant projection factor of the tip-link axis on the hair-bundle displacement axis so that, for a change of position, of the hair bundle *X*, the tip-link extension changes by x=γX. Each of the $N_{\text{t}}$ tip links in the hair bundle contributes a projected force $f_{\text{t}}^{j}$ along the tip-link axis given by

(2) $$f_{\text{t}}^{j}=k_{\text{t}}\left(\gamma \left(X-{\tilde{X}}_{0}\right)-d^{j}\right)\;,$$

where $k_{\text{t}}$ is the tip-link stiffness and $d^{j}$ the varying distance between the tip-link fork and the membrane of the lower stereocilium along the tip-link axis. An illustration of the corresponding geometry is shown in Fig. 2 *A*. The displacement ${\tilde{X}}_{0}$ is a reference position of the *X* axis that sets a reference tension in the tip links for a given hair-bundle position *X* and distance *d*. This tension stems from the action of myosin motors, which incessantly pull on the tip link’s upper insertion point (33). The parameter ${\tilde{X}}_{0}$ is therefore linked to a reference position of these myosin motors. Focusing on shorter timescales than those associated with myosin-motor displacements, we consider ${\tilde{X}}_{0}$ a constant quantity in the following.

To close the system of equations, we now need to determine how the distance $d^{j}$ relates to the hair-bundle displacement *X* for each tip link *j*. Force balance on either of the two channels connected to the tip link reads as follows:

(3) $$k_{\text{t}}\left(\gamma \left(X-{\tilde{X}}_{0}\right)-d\right)=2\frac{d+a\;\text{sin}\;\alpha }{a+d\;\text{sin}\;\alpha }\left[k_{\text{a}}\left(a_{\text{adapt}}-a-\frac{n}{2}\delta \right)-\frac{dV_{\text{b},n}}{da}\right],$$

where the coordinate *a* corresponds to the distance between either of the two anchoring points of the tip link and the tip link’s central axis; α is the angle between the perpendicular to the tip-link axis and the membrane plane; $k_{\text{a}}$ is the adaptation springs’ stiffness; $a_{\text{adapt}}$ sets the inter-channel distance for which the adaptation springs slacken; δ is the single-channel gating swing; n=0, 1, or 2 is the number of open channels in the pair; and $V_{\text{b},n}$ is the elastic potential of the lipid bilayer in which the mechanosensitive channels are embedded, which depends on *n* (Fig. 2 *A*). This elastic membrane potential is derived from the one-dimensional interaction potentials mediated by the membrane between two mechanosensitive channels of large conductance in *Escherichia coli*, as modeled previously (21,30). Following our previous study (20), we mimic the shape of the elastic potentials used in that study with the following analytic expressions:

(4) $$\begin{array}{l} V_{\text{b},0}\left(a\right)=E_{\text{CC}}\left(\frac{a-a_{\text{cross},\text{CC}}}{a_{\text{min}}-a_{\text{cross},\text{CC}}}\right)\text{exp}\left[-{\left(\frac{a-a_{\text{min}}}{l_{\text{v}}}\right)}^{2}\right]\\ V_{\text{b},1}\left(a\right)=E_{\text{OC}}\left[\frac{\left(a_{\text{cross},\text{OC}}-a\right){\left(a_{\text{cross},\text{OC}}-a_{\text{min}}\right)}^{2}}{{\left(a-a_{\text{min}}\right)}^{3}}\right]\text{exp}\left[-{\left(\frac{a-a_{\text{min}}}{l_{\text{v}}}\right)}^{2}\right]\\ V_{\text{b},2}\left(a\right)=E_{\text{OO}}\left(\frac{a-a_{\text{cross},\text{OO}}}{a_{\text{min}}-a_{\text{cross},\text{OO}}}\right)\text{exp}\left[-{\left(\frac{a-a_{\text{min}}}{l_{\text{v}}}\right)}^{2}\right]\;. \end{array}$$

The values of the different parameters entering these expressions are given in Table 1, and a plot is provided in (20). Finally, geometrical constraints impose that *d* and *a* are related by

(5) $$d=\sqrt{l^{2}-{\left(a\;\text{cos}\;\alpha \right)}^{2}}-a\;\text{sin}\;\alpha \;,$$

where *l* is the length of the tip-link fork. A full description of that part of the model and detailed graphical illustrations are given in (20), and additional parameter values used here are provided in Table 1.

**Table 1:** Model parameters

	Parameter	Description	Value	Source
Mechanics	α	angle of adaptation springs with respect to horizontal	15°	
	$K_{\text{sp},0}$	reference combined stiffness of the stereociliary pivots	0.65 mN ·m^−1^	Jaramillo and Hudspeth (34)
	$\gamma_{0}$	reference geometrical scaling factor	0.12	Choe et al. (10)
	$N_{\text{t}}$	number of tip links	$7/8\cdot N_{\text{s}}$	Choe et al. (10)
	$M_{\text{HB}}$	wet mass of the hair bundle	100 pg	Baumgart (32)
	$\xi_{\text{HB}}$	friction coefficient of the hair bundle	$100\;\text{nN}\cdot \text{s}\cdot {\text{m}}^{-1}$	Choe et al. (10)
Elastic membrane potentials	$a_{\text{min}}$	minimal value of *a*	1.5 nm	
	$l_{\text{v}}$	characteristic decay length	1.5 nm	
	$a_{\text{cross},\text{CC}}$	reference value of *a* in the CC state	3 nm	
	$a_{\text{cross},\text{OC}}$	reference value of *a* in the OC state	2.75 nm	All adapted from Ursell et al. (21)
	$a_{\text{cross},\text{OO}}$	reference value of *a* in the OO state	2.5 nm	
	$E_{\text{CC}}$	value of $V_{\text{b},0}$ at $a_{\text{min}}$	−2.5 $k_{\text{B}}T$	
	$E_{\text{OO}}$	value of $V_{\text{b},2}$ at $a_{\text{min}}$	−25 $k_{\text{B}}T$	
Calcium cycle	$E_{\text{Ca}2+}$	energy associated with Ca^2+^ binding/unbinding (closed state)	$1\;k_{\text{B}}T$	adapted from Beurg et al. (35)
	$k_{\text{b}}$	reaction constant of Ca^2+^ binding/unbinding	$1\;{\text{ms}}^{-1}\cdot {\text{μM}}^{-1}$	Choe et al. (10)
	$\bar{k}$	attempt frequency for channel opening and closing	$10\;{\text{ms}}^{-1}$	Beurg et al. (35)
	$K_{\text{d}}$	Ca^2+^ dissociation constant	20μM	Beurg et al. (35)
	${\left[{\text{Ca}}^{2+}\right]}^{\text{closed}}$	Ca^2+^ concentration at the binding site of a closed channel	0.05μM	Choe et al. (10)
	${\left[{\text{Ca}}^{2+}\right]}^{\text{open}}$	Ca^2+^ concentration at the binding site of an open channel	37μM	Choe et al. (10)

### Calcium cycle

Here we describe implementation of the influence of Ca^2+^ binding and unbinding to the channels on their gating states. We hypothesize one Ca^2+^ binding site per channel, which leads to a total of three Ca^2+^-binding states for a given pair of channels, with a total of zero, one, or two ions bound to the pair. As argued above, the rates of Ca^2+^ binding are proportional to the local Ca^2+^ concentration, ${\left[{\text{Ca}}^{2+}\right]}^{\text{open}}$ and ${\left[{\text{Ca}}^{2+}\right]}^{\text{closed}}$ in the open and closed states, respectively. The rate of Ca^2+^ unbinding, however, is independent of the local concentration and, therefore, independent of the channels’ state in our model. Together, this leads to

(6) $$\begin{array}{l} k_{\text{Ca}}^{+,\text{O}}=k_{\text{b}}{\left[{\text{Ca}}^{2+}\right]}^{\text{open}}\\ {\text{k}}_{\text{Ca}}^{+,\text{C}}={\text{k}}_{\text{b}}{\left[{\text{Ca}}^{2+}\right]}^{\text{closed}}\\ {\text{k}}_{\text{Ca}}^{-}={\text{k}}_{\text{b}}{\text{K}}_{\text{d}}\;, \end{array}$$

where $k_{\text{Ca}}^{+,\text{O}}$ and $k_{\text{Ca}}^{+,\text{C}}$ are the rates of Ca^2+^ binding in the open and closed states, respectively, $k_{\text{Ca}}^{-}$ is the rate of Ca^2+^ unbinding, $k_{\text{b}}$ is the reaction constant of Ca^2+^ binding, and $K_{\text{d}}$ is the Ca^2+^ dissociation constant.

### Opening and closing transition rates

To complete the model, we now focus on identifying the probabilities of the different states a channel pair can occupy and how they evolve in time. With physiological parameters, and taking into account the membrane elastic potentials, the energy of the open-closed (OC) state is higher than that of the OO or CC states. In the following, we therefore ignore the occupancy of this state and investigate only the transitions between the OO and CC states. This corresponds to investigating a perfect cooperativity between the two channels, mediated by the lipid bilayer (20). Taking into account the different occupancy states of the channels by Ca^2+^, we are therefore left with a total of six states: three Ca^2+^ binding states for each of the two gating states of the channel pair.

Transitions between each of these adjacent states in this calcium cycle occur with specific rates, represented schematically in Fig. 2 *B*. Among these rates, the Ca^2+^ binding and unbinding rates have already been discussed, and we need to specify the transition rates between the OO and CC states with a given number of bound Ca^2+^ ions. To write these rates, we suppose that each of these transitions goes through its respective OC state, which serves as a limiting transition state. The associated activation energy allows us to write the transition rate using Kramers’ reaction rate theory. We hypothesize that the presence of one calcium ion bound to a closed channel lowers its energy by the fixed amount $E_{\text{Ca}}$. The energies of each of the six states represented in Fig. 2 therefore read

(7) $$\left\{ \begin{array}{l} E_{1}=E_{\text{CC}}\\ E_{2}=E_{\text{CC}}-E_{\text{Ca}}\\ E_{3}=E_{\text{CC}}-2E_{\text{Ca}}\\ E_{4}=E_{5}=E_{6}=E_{\text{OO}}\;, \end{array}\right.$$

where $E_{\text{CC}}$ and $E_{\text{OO}}$ are, respectively, the energies of the CC and OO states in the absence of calcium ions. The energies $E_{1⇌6}$, $E_{2⇌5}$, and $E_{3⇌4}$ of the respective transition states between states 1 and 6, 2 and 5, and 3 and 4 in both directions read

(8) $$\left\{ \begin{array}{l} E_{1⇌6}=E_{\text{OC}}\\ E_{2⇌5}=E_{\text{OC}}-E_{\text{Ca}}\\ E_{3⇌4}=E_{\text{OC}}-E_{\text{Ca}}. \end{array}\right.\;$$

For the transition state between states 2 and 5, there are, a priori, two possibilities: OCa-C and O-CCa. Because we know that the closed state is stabilized by calcium, the intermediate state O-CCa is of lower energy. Because of the exponential dependence of the transition rate on the transition-state energy, we consider that the state O-CCa is strongly favored, leading to the expression given above. Note that we assume that the binding of Ca^2+^ does not affect the energy of the open state. As a result, the height of the energy barrier in the transitions 5→2 and 4→3 is lowered by $E_{\text{Ca}}$ in both cases compared with that of the transition 6→1. The energy barriers in the opposite directions, however, behave differently; the barrier of the transition 2→5 is unchanged compared with that of 1→6, and that of 3→4 is increased by $E_{\text{Ca}}$. Given these energies, Kramers’ rate theory allows us to write the following expressions for the forward and reverse transition rates between states 1 and 6, 2 and 5, and 3 and 4:

(9) $$\left\{ \begin{array}{l} k_{1\to 6}=\bar{k}\;{\text{e}}^{-\beta \left(E_{\text{OC}}-E_{\text{CC}}\right)}\\ k_{6\to 1}=\bar{k}\;{\text{e}}^{-\beta \left(E_{\text{OC}}-E_{\text{OO}}\right)}\\ k_{2\to 5}=\bar{k}\;{\text{e}}^{-\beta \left(E_{\text{OC}}-E_{\text{CC}}\right)}\\ k_{5\to 2}=\bar{k}\;{\text{e}}^{-\beta \left(E_{\text{OC}}-E_{\text{OO}}-E_{\text{Ca}}\right)}\\ k_{3\to 4}=\bar{k}\;{\text{e}}^{-\beta \left(E_{\text{OC}}-E_{\text{CC}}+E_{\text{Ca}}\right)}\\ k_{4\to 3}=\bar{k}\;{\text{e}}^{-\beta \left(E_{\text{OC}}-E_{\text{OO}}-E_{\text{Ca}}\right)}\;, \end{array}\right.$$

where $\beta =1/\left(k_{\text{B}}T\right)$ is the thermodynamic *beta* or inverse thermal energy, with $k_{\text{B}}$ the Boltzmann constant and *T* the temperature, and where $\bar{k}$ is a pre-exponential factor called “the attempt frequency” that sets a baseline rate to these transitions.

### Probability dynamics

We can now write the time-evolution equations for the occupancy probabilities of each of the six different states shown in Fig. 2:

(10) $$\left\{ \begin{array}{l} {\dot{p}}_{1}=\;2k_{\text{Ca}}^{-}e^{-\beta E_{\text{Ca}}}p_{2}+\bar{k}e^{-\beta \left(E_{\text{OC}}-E_{\text{OO}}\right)}p_{6}-\left[\bar{k}e^{-\beta \left(E_{\text{OC}}-E_{\text{CC}}\right)}+k_{\text{Ca}}^{+,\text{C}}e^{\beta E_{\text{Ca}}}\right]p_{1}\\ {\dot{p}}_{2}=\;\bar{k}e^{-\beta \left(E_{\text{OC}}-E_{\text{OO}}-E_{\text{Ca}}\right)}p_{5}+\frac{1}{2}k_{\text{Ca}}^{-}e^{-\beta E_{\text{Ca}}}p_{3}+\frac{1}{2}k_{\text{Ca}}^{+,\text{C}}e^{\beta E_{\text{Ca}}}p_{1}-\left[\bar{k}e^{-\beta \left(E_{\text{OC}}-E_{\text{CC}}\right)}+k_{\text{Ca}}^{+,\text{C}}e^{\beta E_{\text{Ca}}}+k_{\text{Ca}}^{-}e^{-\beta E_{\text{Ca}}}\right]p_{2}\\ {\dot{p}}_{3}=\;2k_{\text{Ca}}^{+,\text{C}}e^{\beta E_{\text{Ca}}}p_{2}+\bar{k}e^{-\beta \left(E_{\text{OC}}-E_{\text{OO}}-E_{\text{Ca}}\right)}p_{4}-\left[\bar{k}e^{-\beta \left(E_{\text{OC}}-E_{\text{CC}}+E_{\text{Ca}}\right)}+k_{\text{Ca}}^{-}e^{-\beta E_{\text{Ca}}}\right]p_{3}\\ {\dot{p}}_{4}=\;2k_{\text{Ca}}^{+,\text{O}}p_{5}+\bar{k}e^{-\beta \left(E_{\text{OC}}-E_{\text{CC}}+E_{\text{Ca}}\right)}p_{3}-\left[\bar{k}e^{-\beta \left(E_{\text{OC}}-E_{\text{OO}}-E_{\text{Ca}}\right)}+k_{\text{Ca}}^{-}\right]p_{4}\\ {\dot{p}}_{5}=\;\frac{1}{2}k_{\text{Ca}}^{+,\text{O}}p_{6}+\frac{1}{2}k_{\text{Ca}}^{-}p_{4}+\bar{k}e^{-\beta \left(E_{\text{OC}}-E_{\text{CC}}\right)}p_{2}-\left[k_{\text{Ca}}^{-}+k_{\text{Ca}}^{+,\text{O}}+\bar{k}e^{-\beta \left(E_{\text{OC}}-E_{\text{OO}}-E_{\text{Ca}}\right)}\right]p_{5}\\ {\dot{p}}_{6}=\;2k_{\text{Ca}}^{-}p_{5}+\bar{k}e^{-\beta \left(E_{\text{OC}}-E_{\text{CC}}\right)}p_{1}-\left[\bar{k}e^{-\beta \left(E_{\text{OC}}-E_{\text{OO}}\right)}+k_{\text{Ca}}^{+,\text{O}}\right]p_{6}\;. \end{array}\right.$$

Note that the prefactors 2 and 1/2 that appear in some terms are due to the fact that the states 2 and 5 are degenerate; in both instances, a single Ca^2+^ ion is free to bind to either of the two available channels, with the same resultant energy in either case.

### Expression of the energies

In the system of Eq. 10, the energies $E_{\text{OO}}$, $E_{\text{OC}}$, and $E_{\text{CC}}$ correspond to the energies of one pair of channels in the respective configurations. These energies include the elastic contributions from the extensions of the tip link and adaptation springs, that of the lipid-bilayer deformations $V_{\text{b},n}$, as well as the gating energy of the channels $E_{\text{g}}$, the energy difference between the open and closed states of a single channel. We define the following energy function for a pair of n=0, 1, or 2 open channels, located a distance of 2a apart, with the hair bundle being positioned at *X*:

(11) $$E\left(a,X,n\right)=2\times \frac{k_{\text{a}}}{2}{\left(a_{\text{adapt}}-a-\frac{n}{2}\delta \right)}^{2}\text{Θ}\left(a_{\text{adapt}}-a-\frac{n}{2}\delta \right)+\frac{k_{\text{t}}}{2}{\left(\gamma \left(X-{\tilde{X}}_{0}\right)-d\right)}^{2}\;\text{Θ}\left(\gamma \left(X-{\tilde{X}}_{0}\right)-d\right)+V_{\text{b},n}\left(a\right)+nE_{\text{g}}\;.$$

Here, *d* is a function of the half inter-channel distance *a* because of the geometric relation Eq. 5, and Θ is the Heaviside step function that represents slacking because neither the tip links nor the adaptation springs are supposed to resist compression. The energies $E_{\text{OO}}$, $E_{\text{OC}}$, and $E_{\text{CC}}$ in Eq. 10 equal E(a,X,n) as given in Eq. 11 with n=0, 1, or 2, respectively.

As seen in Eq. 3, together with Eq. 5, the position of the hair bundle’s tip *X* is a function of the half inter-channel distance *a* as well as of the number *n* of open channels in the pair via the elastic membrane potential $V_{\text{b},n}$. We therefore have three different relations X(a): one per gating state of the pair (OO, OC, or CC). Each of these relations is invertible so that we can write the equations in terms of the sole variable *X*, replacing the variable *a* in state *n* in Eq. 11 by the corresponding solution to Eq. 3, $a_{n}\left(X\right)$. Finally, we obtain expressions that can be summarized under the form

(12) $$\left\{ \begin{array}{l} E_{\text{CC}}\left(X\right)=E\left(a_{0}\left(X\right),X,0\right)\\ E_{\text{OC}}\left(X\right)=E\left(a_{1}\left(X\right),X,1\right)\\ E_{\text{OO}}\left(X\right)=E\left(a_{2}\left(X\right),X,2\right)\;. \end{array}\right.$$

### Closing the system

Considering that Eq. 1 is second order, we can split it into two first-order equations by introducing the variable $Y=\dot{X}$. Doing so, we use the vector $S={\left(X,Y,p_{1},p_{2},p_{3},p_{4},p_{5},p_{6}\right)}^{\text{T}}$ to represent the state of the system at any given time, where the symbol T corresponds to the matrix transposition operation. The whole model now reads

(13) $$\dot{S}=F\left(S\right)\;,$$

where the vectorial function F corresponds to Eq. 1 and Eq. 10 together with the other equations given above that allow us to express F as a function of the components of S only.

### Parameter values

Several of the model parameters can be estimated from the literature. We list in Table 1 the parameters that relate to the mechanics of the hair bundle, that of the elastic deformations of the membrane, and to the calcium cycle. The parameters that do not appear in Table 1 are the same as the default values in (20). The parameter ${\tilde{X}}_{0}$ entering Eq. 2 is not directly observable from experiments because it is linked to the position of myosin motors within stereocilia. To infer its value, we first choose that the origin of the *X* axis (X=0) is such that it is a resting point of the hair bundle, i.e., a static solution of Eq. 1. This fixes the value of $X_{\text{sp}}$, the resting position of the stereociliary pivots relative to the origin, provided that we know all other parameters in that equation. To determine the value of ${\tilde{X}}_{0}$, we impose in addition that, at X=0, the steady-state open probability of the channels $P_{\text{open}}=p_{4}+2p_{5}+p_{6}$ equals 0.15. This value corresponds to the resting open probability of mammalian inner hair cells tuned to kilohertz frequencies (36, 37, 38, 39).

The parameter $M_{\text{HB}}$ represents the effective mass of the hair bundle as it moves in liquid. The total mass of stereocilia in a hair bundle can be estimated from the bundle’s geometry and the actin content of stereocilia. This leads to a “dry mass” of about 50–60 pg (8,40). However, hair bundles are immersed in liquid. As they oscillate, they drag along an additional volume of fluid located within the boundary layer, effectively increasing the apparent hair bundle’s mass (41). Such a “wet mass” has been estimated using a detailed finite-element model of the hair bundle and its surrounding fluid to be on the order of 100 pg (32).

## Results

Having all parameters at hand, we perform linear stability analysis around the fixed point X=0 on the dynamics given by Eq. 13. In the following figures, we plot the real and imaginary components of the pair of eigenvalues with maximum real part, which characterize the dynamics of our system in the proximity of the steady state. Because this pair of complex-conjugated eigenvalues crosses the imaginary axis, the real part changes sign, and an oscillatory decay becomes a limit-cycle oscillation, corresponding to a Hopf bifurcation (6). All other eigenvalues have negative real parts. They correspond to modes that relax on a finite timescale at the bifurcation. More generally, looking at the long-time dynamics of the hair bundle, only the pair of eigenvalues of largest real component controls its behavior.

We start by investigating the dependence of the eigenvalues on the number of stereocilia $N_{\text{s}}$ in the hair bundle. Along the tonotopic axis of the cochlea, hair cells tuned to higher frequencies have more stereocilia than those tuned to lower frequencies, and their stereocilia tend to be shorter, which makes their hair bundles stiffer (42, 43, 44). In addition, the total mass of actin contained in each hair bundle of a given cochlea has been shown to be roughly constant along the chicken cochlea (45). This suggests a relationship between the geometric gain factor γ, the collective stereociliary pivot stiffness $K_{\text{sp}}$, and the number of stereocilia in the bundle $N_{\text{s}}$ (10). Specifically, we follow (10) and assume that $\gamma \propto N_{\text{s}}$ and $K_{\text{sp}}\propto N_{\text{s}}^{3}$, reducing the number of free parameters in the model. We postulate, therefore, that $K_{\text{sp}}=K_{\text{sp},0}\cdot {\left(N_{\text{s}}/50\right)}^{3}$ and $\gamma =\gamma_{0}\cdot \left(N_{\text{s}}/50\right)$, where $K_{\text{sp},0}$ and $\gamma_{0}$ are a reference stiffness of the stereociliary pivots and a reference scaling factor in a hair bundle with 50 stereocilia. Their values are set to correspond to those used in (20) (Table 1).

**Figure 3:**
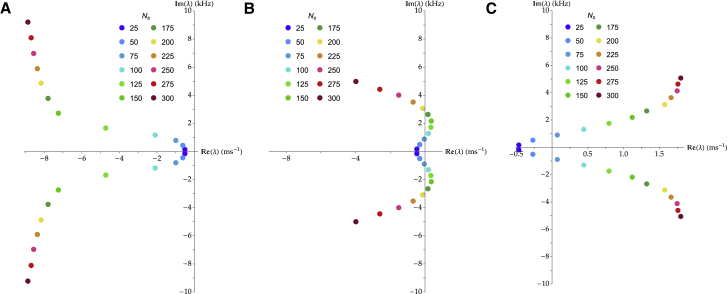
Dependence of the pair of eigenvalues λ with the largest real part on the number of stereocilia $N_{\text{s}}$, under the constant actin-mass hypothesis and for three values of the attempt frequency $\bar{k}$. (*A*) For $\bar{k}=\;1\cdot 10^{3}\;{\text{s}}^{-1}$, the hair bundle displays only damped oscillations. (*B*) For $\bar{k}=1\cdot 10^{4}\;{\text{s}}^{-1}$, the hair bundle shows limit-cycle oscillations for a limited range of values of $N_{\text{s}}$, here roughly between 50 and 200. (*C*) For $\bar{k}=5\cdot 10^{4}\;{\text{s}}^{-1}$, the hair bundle presents limit-cycle oscillations above a threshold value of $N_{\text{s}}$, here around 50. To see this figure in color, go online.

We investigate the dependence of the characteristic frequency of the modeled hair bundle on $N_{\text{s}}$, following the constant actin-mass hypothesis described above, to compare our results with those of (10). We plot in Fig. 3 this dependence for three values of the attempt frequency $\bar{k}$. For small values of $\bar{k}$, the hair bundle remains quiescent, regardless of the number of stereocilia $N_{\text{s}}$ (Fig. 3 *A*). For large values of $\bar{k}$, the hair bundle oscillates above a critical value of $N_{\text{s}}$ (Fig. 3 *C*). At intermediate values of $\bar{k}$, the hair bundle oscillates within a limited range of $N_{\text{s}}$ values (Fig. 3 *B*). As $N_{\text{s}}$ is varied, $\gamma \propto N_{\text{s}}$ and $K_{\text{sp}}\propto N_{\text{s}}^{3}$. As a result, in Eq. 1, the pivoting stiffness term scales as $N_{\text{s}}^{3}$ and the tip-link force term scales as $N_{\text{s}}^{2}$ because each individual tip-link force is set by Eq. 3, of which the right side is independent of $N_{\text{s}}$. Therefore, for small values of $N_{\text{s}}$, γ is small, and the combined tip-link forces through which the calcium cycle powers the system become small so that the hair-bundle motion is dominated by its passive contributions. For large values of $N_{\text{s}}$, the pivoting-stiffness term becomes dominant, and we are driven back toward a passive behavior. As in (10), we obtain characteristic frequencies that increase with the number of stereocilia $N_{\text{s}}$.

### Calcium dependency

**Figure 4:**
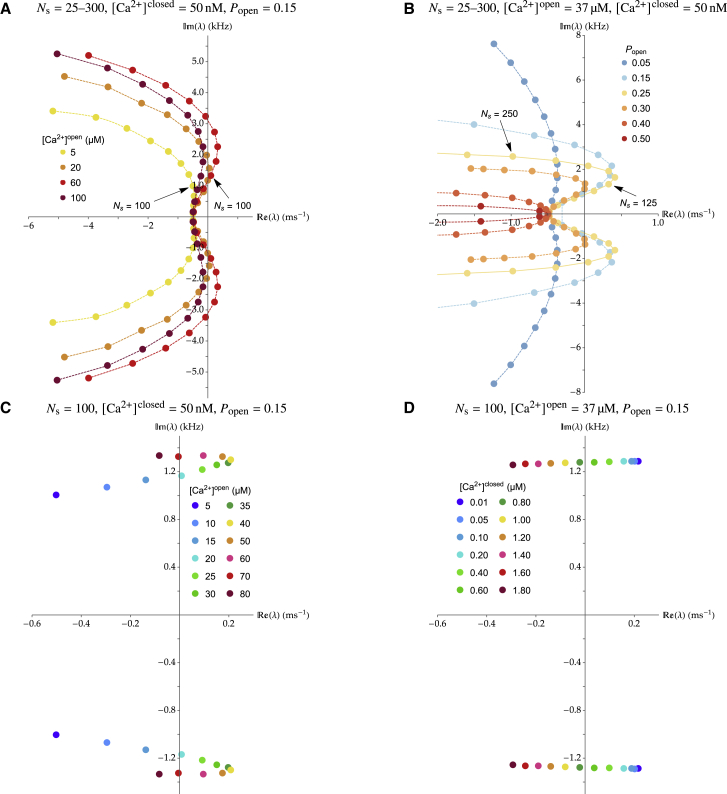
Dependence of the most unstable pair of eigenvalues λ on the number of stereocilia $N_{\text{s}}$, the calcium concentration in the vicinity of the channel in the O state ${\left[{\text{Ca}}^{2+}\right]}^{\text{open}}$ and in the C state ${\left[{\text{Ca}}^{2+}\right]}^{\text{closed}}$, and the open probability at rest $P_{\text{open}}$. (*A*) The eigenvalues are plotted in four families of curves, each corresponding to a different value of ${\left[{\text{Ca}}^{2+}\right]}^{\text{open}}$, while varying the number of stereocilia $N_{\text{s}}$ from 25 to 300 by steps of 25 as in Fig 3 and at fixed $\bar{k}=10^{4}\;{\text{s}}^{-1}$, [Ca^2+^]^closed^ = 50 nM and $P_{\text{open}}=0.15$. (*B*) The eigenvalues are plotted similarly as in (*A*), in six families of curves, each corresponding to a different value of $P_{\text{open}}$, for ${\left[{\text{Ca}}^{2+}\right]}^{\text{open}}=37\;\mu \text{M}$ and with the same values of $\bar{k}$, ${\left[{\text{Ca}}^{2+}\right]}^{\text{closed}}$, and varied $N_{\text{s}}$ as in (*A*). (*C* and *D*) The eigenvalues are plotted for various calcium concentrations ${\left[{\text{Ca}}^{2+}\right]}^{\text{open}}$ (*C*) and ${\left[{\text{Ca}}^{2+}\right]}^{\text{closed}}$ (*D*) at fixed $N_{\text{s}}=100$, $\bar{k}=10^{4}\;{\text{s}}^{-1}$, and $P_{\text{open}}=0.15$. To see this figure in color, go online.

Hair-bundle oscillations are known to be affected by the concentration of calcium in the surrounding milieu (8). In Fig. 4, we show the dependence of the eigenvalues on calcium concentration at the binding site when the channels are open or closed, that is varying the parameters ${\left[{\text{Ca}}^{2+}\right]}^{\text{open}}$ and ${\left[{\text{Ca}}^{2+}\right]}^{\text{closed}}$ as well as on the open probability of the channels at steady state $P_{\text{open}}$. We display in Fig. 4 *A* the dependence of the eigenvalues as a function of the number of stereocilia $N_{\text{s}}$ for four different values of the calcium concentration in the vicinity of the channel in the open state ${\left[{\text{Ca}}^{2+}\right]}^{\text{open}}$. We see that the system transitions from damped to limit-cycle and then back to damped oscillations as ${\left[{\text{Ca}}^{2+}\right]}^{\text{open}}$ increases, within a given range of $N_{\text{s}}$ values, here between 100 and 200 for ${\left[{\text{Ca}}^{2+}\right]}^{\text{open}}=60\;\text{μM}$. The system displays limit-cycle oscillations for calcium concentrations in a window around 40 μM. For lower and higher values of ${\left[{\text{Ca}}^{2+}\right]}^{\text{open}}$, the eigenvalues are confined to the left of the imaginary axis, in the regimen of damped oscillations, for all $N_{\text{s}}$ values. This can be understood from the necessity of having a cycle between the six states of Fig. 2 *B*. At large ${\left[{\text{Ca}}^{2+}\right]}^{\text{open}}$, the system is too biased toward states 3 and 4 via the dependence of $k_{\text{Ca}}^{+,\text{O}}$ in ${\left[{\text{Ca}}^{2+}\right]}^{\text{open}}$. If ${\left[{\text{Ca}}^{2+}\right]}^{\text{open}}$ is too low, however, the opposite happens, and most channels have no Ca^2+^ bound. The evolution of the eigenvalues with ${\left[{\text{Ca}}^{2+}\right]}^{\text{open}}$ at fixed $N_{\text{s}}=100$ is shown in Fig. 4 *C*. We can see that the variation in characteristic frequency remains relatively small, with a slight increase toward large ${\left[{\text{Ca}}^{2+}\right]}^{\text{open}}$ values. This stems from a higher $k_{\text{Ca}}^{+,\text{O}}$ rate with larger ${\left[{\text{Ca}}^{2+}\right]}^{\text{open}}$ values.

Because the effect of calcium binding on hair-bundle oscillations depends, at any given time, on the total number of open channels, we expect the behavior of the hair bundle to change as the value of the open probability at steady state is varied. In Fig. 4 *B*, we show the eigenvalues’ dependence on $N_{\text{s}}$, similar to Fig. 4 *A*, for six different values of $P_{\text{open}}$. With the parameter values given in Table 1, the hair bundle displays limit-cycle oscillations within a limited range of $P_{\text{open}}$ values, from about 0.15 to about 0.30. The existence of a range of values of $P_{\text{open}}$ for which oscillations occur can be interpreted qualitatively as follows. Varying $P_{\text{open}}$ affects the value of ${\tilde{X}}_{0}$, which sets the value of tension in the tip links via Eq. 2. This, in turn, affects the energies via Eq. 11, which further enter the system of Eq. 10 in exponential functions. The resulting steady state corresponds to relative occupancies that follow sigmoidal-type laws in the energies. If the values of these energies are too biased in a particular direction, then the energy $E_{\text{Ca}}$ gained by Ca^2+^ binding is not large enough to sufficiently affect the state occupancies and to power spontaneous oscillations. That illustrates why there is a range of $P_{\text{open}}$ values for which oscillations occur. In addition, this range is biased toward values of $P_{\text{open}}$ smaller than 1/2 because, in the system of Eq. 10, not all transition rates are affected equally by the ${\text{Ca}}^{2+}$ binding energy $E_{\text{Ca}}$. The transitions between the open states 6, 5, and 4 are not affected by this energy $E_{\text{Ca}}$, whereas those between the closed states 1, 2, and 3 are affected by $E_{\text{Ca}}$. Therefore, the ${\text{Ca}}^{2+}$ binding energy is used most efficiently when the open states 6, 5, and 4 are relatively depopulated compared with states 1, 2, and 3, corresponding to an overall open probability of less than 0.5.

In this description, we used constant values of the local Ca^2+^ concentrations ${\left[{\text{Ca}}^{2+}\right]}^{\text{open}}$ and ${\left[{\text{Ca}}^{2+}\right]}^{\text{closed}}$ in the open and closed states of the channel, respectively, which is valid only when the local Ca^2+^ concentration adjusts fast enough to the channel’s state. In a hemi-infinite space, the evolution of the Ca^2+^ concentration a given distance from a point source, such as a mechanotransduction channel, is given by Eq. 2.9 (when open) or 2.12 (when closed) of (46), up to a factor two stemming from symmetry arguments, as in (47). Hypothesizing a Ca^2+^-binding site located intracellularly 5 nm away from the channel pore (10) and a Ca^2+^ diffusion coefficient of $8\cdot 10^{-8}$ m^2^ s^−1^ (48), and with our default extracellular and intracellular steady-state Ca^2+^ concentrations given in Table 1, we obtain that the Ca^2+^ concentration reaches 95% of its maximal value ${\left[{\text{Ca}}^{2+}\right]}^{\text{open}}$ only 5 μs after channel opening to then drop within 100 μs to about 60 nM, less than 0.2% of that value. Similar estimates are made in (10). To investigate whether this larger falling time of the Ca^2+^ concentration in the closed state can have an effect on our results, we investigate in Fig. 4 *D* the dependence of the system’s eigenvalues as a function of an effective Ca^2+^ concentration in the closed state, considered to be an interpolation between the different values of the local Ca^2+^ concentration after the channel has closed and before it reopens. We can see that the eigenvalues are hardly changed when this effective Ca^2+^ concentration is varied between 10 and 100 nM, a value reached about 30 μs after channel closure. This analysis demonstrates that, to study hair-bundle oscillations up to 10 kHz or even slightly more, we can safely ignore the rising and falling times in the local Ca^2+^ concentration at the binding site and consider this to be equal to its steady-state values ${\left[{\text{Ca}}^{2+}\right]}^{\text{open}}$ and ${\left[{\text{Ca}}^{2+}\right]}^{\text{closed}}$, when the channel is in the open or closed state, respectively. As expected, increasing ${\left[{\text{Ca}}^{2+}\right]}^{\text{closed}}$ eventually causes the oscillations to cease because of Ca^2+^ rebinding to the channel before it reopens (10). Because the Ca^2+^ binding site is thought to be located only within several nanometers from the channel pore, Ca^2+^ buffers do not play a role in the process we describe because they do not affect the Ca^2+^ concentration at the binding site on the relevant timescales. The situation would change, however, if the binding site were located at significantly greater distances from the channel. Our conclusion here agrees with the results reported in (49), where it is shown that, at distances from the channel shorter than the buffer “length constant”—which for BAPTA is about 30 nm and for EGTA about 420 nm—the calcium concentration behaves as it would in an unbuffered scenario.

**Figure 5:**
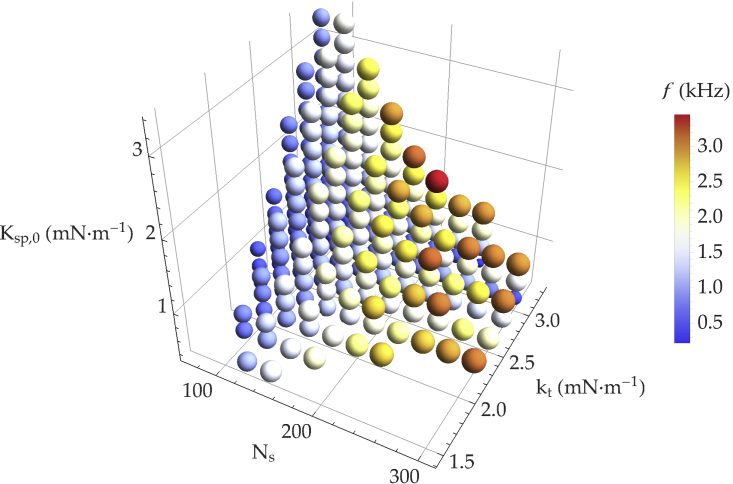
4D plot of the parameter values for which the system shows limit-cycle oscillations as a function of $k_{\text{t}}$, $N_{\text{s}}$, and $K_{\text{sp},0}$, with $\bar{k}=10^{4}\;{\text{s}}^{-1}$, $P_{\text{open}}=0.15$, ${\left[{\text{Ca}}^{2+}\right]}^{\text{open}}=37\;\mu \text{M}$, and ${\left[{\text{Ca}}^{2+}\right]}^{\text{closed}}=50\;\text{nM}$. Each plotted bubble corresponds to an oscillatory state. As the corresponding frequency varies from 0.2 kHz to 3.5 kHz, the color of the bubbles changes from blue to red, and their size increases. To see this figure in color, go online.

Finally, in Fig. 5, we represent the frequencies of the spontaneous oscillations, when they exist, in three dimensions as a function of the parameters $K_{\text{sp},0}$, $N_{\text{s}}$, and $k_{\text{t}}$, at fixed $P_{\text{open}}$, ${\left[{\text{Ca}}^{2+}\right]}^{\text{open}}$, and ${\left[{\text{Ca}}^{2+}\right]}^{\text{closed}}$. Each bubble corresponds to an oscillatory state associated with a unique triplet of these parameters. Its color and size code for the frequency of the corresponding spontaneous oscillations.

## Discussion

In our previous work, we developed a new gating-spring model with cooperative channels (20) and then used it to explain changes observed in the biophysical properties of hair cells during development and tip-link regeneration (50). In the present study, we developed the model further and show how the mechanotransduction apparatus of a hair cell can not only transduce but also amplify incident sounds at kilohertz frequencies, typical of amniote hearing. Specifically, we investigated whether, within this framework, the electrochemical gradient of Ca^2+^ can power spontaneous oscillations in hair cells at such frequencies.

The proposal that the electrochemical gradient of Ca^2+^ ions can power the active process through their binding and unbinding to the mechanosensitive channels is not new. Our work was particularly influenced by Choe et al. (10) showing that a Hopf bifurcation can arise from the Ca^2+^-dependent fast adaptation mechanism without the need for myosin motor activity. There are, however, major differences between the two models. In the work by Choe et al. (10), all the “forward” rate constants were identical, as were their symmetric “reverse” rate constants. Taking different values for these two rate constants therefore drove the cycle of hair-bundle oscillations. Here, instead, the rate constants are based on Kramers’ rate theory, using the OC state of the channel pair as the activation state. The oscillations are driven by the electrochemical gradient of Ca^2+^, as described by the out-of-equilibrium rates of Ca^2+^ binding and unbinding in Eq. 6. The second main difference is that here, a single calcium binding site per channel suffices to give rise to spontaneous oscillations, whereas two binding sites were required in the work by Choe et al. (10) for energetic and kinetic considerations. Finally, because of the mobility of the channels within the lipid membrane, together with a difference in membrane elastic energies between the OO and CC states of the channel pair, our model is capable of producing spontaneous oscillations without requiring a large individual-channel gating swing. It relies instead chiefly on the state-dependent changes in the thickness of the hydrophobic core of the channel at the interface with the lipid bilayer, resulting in mutual attraction or repulsion of the channels at short distances (20,21,30).

Our results are consistent with a number of experimental findings. First, the frequency of spontaneous oscillations increases with the number of stereocilia, in agreement with the tonotopic arrangement along the cochlea (44,45). Second, Tobin et al. (44) showed that all three parameters, $N_{\text{s}}$, $k_{\text{t}}$, and $K_{\text{sp}}$, increase their values along the tonotopic axis of the cochlea. This is in agreement with the results shown in Fig. 5, where the characteristic frequency of oscillations increases as these parameters are increased simultaneously. Third, spontaneous oscillations in the model arise only in a limited range of Ca^2+^ concentration. With physiological parameters, this range is approximately 20–70 μM, in agreement with the Ca^2+^ concentration of the cochlear endolymph (51). The results show a similar trend as a function of the open probability at rest, for which we observe spontaneous oscillations between approximately 0.10 and 0.35. This range corresponds to the open probabilities of the mechanotransduction channels in mammalian inner hair cells as well as in hair cells of some lizard species (38,39,52). Therefore, given the shape of the sigmoidal open-probability versus displacement curve, a high-frequency sinusoidal stimulus results in a non-zero integral voltage response at such open probabilities (38). We conclude from these results that the range of resting open probabilities characteristic of these hair cells not only maximizes the integrated voltage response for a given high-frequency input but is also compatible with mechanical amplification.

In the experimentally observed and extensively modeled low-frequency oscillations of hair cells from the bullfrog sacculus, myosin motors play a direct role in the oscillations (53,54). The hair-bundle negative stiffness and the motor-based slow adaptation together engender large-amplitude oscillations in those vestibular hair cells tuned to frequencies of several hertz to tens of hertz (8,53). During a complete cycle of these relaxation oscillations, the channels’ open probability varies extensively, from almost zero to about one. In contrast, myosin motors do not play a direct role in the amplification mechanism proposed here. Instead, their role is implicitly limited to setting the channels’ open probability at steady state to a specific value, which they are known to do by means of a process called “slow adaptation” (4,55). Our results indicate that large-amplitude oscillations are neither expected from the proposed amplification mechanism nor required to support the active process in general. Sufficiently close to the instability threshold of a supercritical Hopf bifurcation, the oscillation amplitude is arbitrarily small, and the open probability can remain near its target value at all times. This could be the reason why, experimentally, one has never observed spontaneous oscillations in cochlear hair cells tuned to frequencies in the kilohertz range. It is possible that spontaneous oscillations in these cells remain so small that they are practically unobservable because of Brownian motion and measurement noise. Therefore, despite the absence of any direct experimental evidence so far, it is possible that auditory hair cells act as cochlear amplifiers at kilohertz frequencies through the mechanism described in this study.

## Author contributions

A.S.K. conceived and designed the project. T.R. and A.S.K. directed the project. E.D. and T.R. designed the model. F.G., B.H., E.D., and T.R. wrote the code. F.G. produced the results and the figures. F.G., T.R., and A.S.K. interpreted the results and wrote the paper. A.S.K. and T.R. contributed equally to this project.
